# Uptake of Remote Physiologic Monitoring in the US Medicare Program: A Serial Cross-sectional Analysis

**DOI:** 10.2196/46046

**Published:** 2023-05-01

**Authors:** Jeffrey R Curtis, James Willig

**Affiliations:** 1 Division of Clinical Immunology and Rheumatology University of Alabama at Birmingham Birmingham, AL United States; 2 Division of Infectious Diseases University of Alabama at Birmingham Birmingham, AL United States

**Keywords:** remote patient monitoring, remote therapeutic monitoring, digital health, mobile technology, mHealth, mobile health, remote monitoring, patient monitoring, cost, economic, payment, Medicare, insurance, health coverage, outpatient

## Introduction

The opportunity to provide continuous care to patients between office visits using digital technologies holds tremendous potential to improve health care quality and patient outcomes. In 2019, the Center for Medicare and Medicaid Services (CMS) launched the remote physiologic monitoring (RPM) program that provided reimbursement for using technology to monitor patients between visits [1]. RPM delivers continuous or periodic digital data to a central location. These data typically are reviewed by clinical staff (eg, nurses, medical assistants) whose time is billed “incident to” the supervising physician. RPM offers an intuitive complement to remote care delivered via telehealth. In part related to the COVID-19 public health emergency, RPM subsequently was expanded by CMS to improve coverage and reduce barriers to access.

The RPM program requires that a biosensor be used to monitor patients between visits, often but not always in conjunction with a smartphone app. For many health conditions, a biosensor device is a logical component to chronic disease management. Examples include continuous glucose monitoring (diabetes), daily weights via a smart scale (heart failure), dysrhythmia detection (cardiac conditions), and ambulatory blood pressure monitoring (hypertension). Early evidence supporting RPM use appears favorable [2]. Outside of isolated published examples that have largely been confined to a single chronic illness, the extent to which RPM has been deployed on a national scale is unknown. Using US Medicare data, we examined the uptake of RPM in the United States from 2019 (its inception year) to 2021.

## Methods

We examined publicly available Medicare Part B National Summary Data File data from January 2019 to December 2021 [3]. We extracted Medicare payment amounts and the associated services allowed based on relevant Current Procedural Terminology (CPT) codes; individual patient information is not available in this data source. RPM services were grouped as setup (CPT 99453), data transmission (CPT 99454), and monitoring time, which is billed in 20-minute increments (CPT 99457,99458). Results were stratified by calendar year and analyzed in R version 4.2.3 (R Foundation for Statistical Computing).

## Results

**Figure 1:**
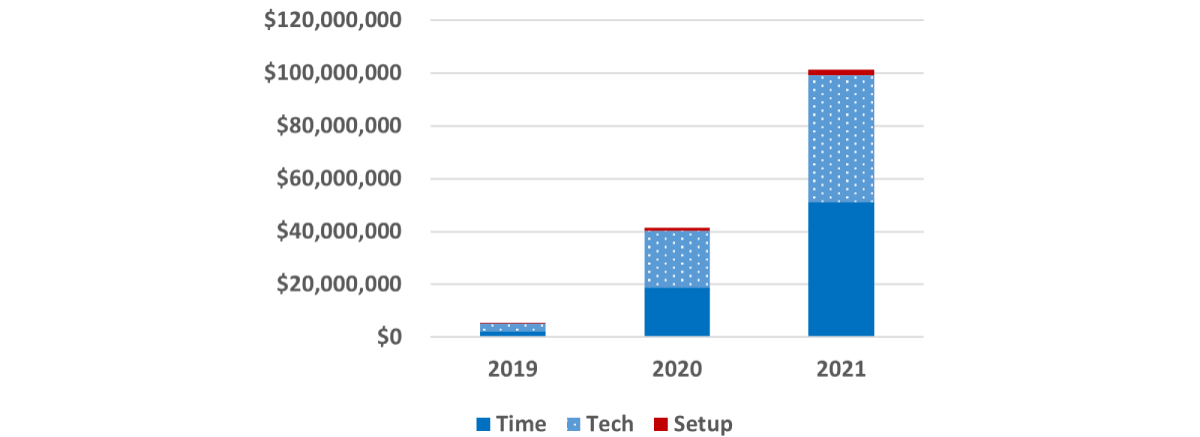
Medicare payments for remote patient monitoring services, 2019-2021.

In 2019, the total amount paid by CMS for RPM was US $5.5 million. In 2020, RPM payments increased almost 9-fold to US $41.5 million, followed by a further 2.5-fold increase in 2021, totaling more than US $101 million annually (Figure 1).

Assuming providers initiate the program and bill setup fees only once per patient, the number of new patients increased from 20,640 (2019) to 90,149 (2020) and further increased to 123,476 (2021). The total payments made by CMS for the technical service (data transmission) were comparable to the payment for the time spent monitoring. Most (69%) monthly reimbursement for patient monitoring was for 20 minutes; only 31% was for monitoring beyond 20 minutes.

## Discussion

Based on national data from the Medicare program, RPM grew approximately 19-fold over 3 years, suggesting rapid uptake. However, some have raised concerns about the potential for overuse of RPM without clinical benefit [3]. Moreover, the use of RPM appears to be confined to a small group of physicians, predominantly primary care providers focused on hypertension or diabetes management [4]. In addition to Medicare, both commercial insurance programs and many states’ Medicaid programs also cover RPM services [4,5]. Importantly, in 2022 CMS further expanded remote monitoring for certain medical specialties (musculoskeletal [rheumatology, orthopedics], respiratory medicine). Under this new program called remote therapeutic monitoring (RTM), a software app alone can be used for monitoring, and patients provide data through the app without a biosensor [6]. The software itself is the medical device and would be registered and cleared by the US Food and Drug Administration as a class 1 (or higher) device.

Thus beginning in 2022, RTM widens the spectrum of health domains available for monitoring, since any patient-reported outcome (eg, disease activity) or clinically relevant information (eg, medication adherence) now is reimbursable. We eagerly await the evaluation of the impact on patient outcomes offered by RPM and RTM, recognizing that much work to optimize their use remains.

## Abbreviations

CMS: Center for Medicare and Medicaid Services
CPT: Current Procedural Terminology
RPM: remote physiologic monitoring
RTM: remote therapeutic monitoring

## Data Availability

Data can be shared and are publicly available (see manuscript for source).
